# Tackling Cancer Stem Cells via Inhibition of EMT Transcription Factors

**DOI:** 10.1155/2016/5285892

**Published:** 2016-10-20

**Authors:** Megan Mladinich, Diane Ruan, Chia-Hsin Chan

**Affiliations:** ^1^Graduate Program in Molecular and Cellular Biology, Stony Brook University, Stony Brook, NY 11790, USA; ^2^Department of Pharmacological Sciences, Stony Brook University, Stony Brook, NY 11794, USA; ^3^Stony Brook Cancer Center, Stony Brook University, Stony Brook, NY 11794, USA

## Abstract

Cancer stem cell (CSC) has become recognized for its role in both tumorigenesis and poor patient prognosis in recent years. Traditional therapeutics are unable to effectively eliminate this group of cells from the bulk population of cancer cells, allowing CSCs to persist posttreatment and thus propagate into secondary tumors. The therapeutic potential of eliminating CSCs, to decrease tumor relapse, has created a demand for identifying mechanisms that directly target and eliminate cancer stem cells. Molecular profiling has shown that cancer cells and tumors that exhibit the CSC phenotype also express genes associated with the epithelial-to-mesenchymal transition (EMT) feature. Ample evidence has demonstrated that upregulation of master transcription factors (TFs) accounting for the EMT process such as Snail/Slug and Twist can reprogram cancer cells from differentiated to stem-like status. Despite being appealing therapeutic targets for tackling CSCs, pharmacological approaches that directly target EMT-TFs remain impossible. In this review, we will summarize recent advances in the regulation of Snail/Slug and Twist at transcriptional, translational, and posttranslational levels and discuss the clinical implication and application for EMT blockade as a promising strategy for CSC targeting.

## 1. Introduction

Cancer stem cells (CSCs) are characterized by their self-renewal and pluripotent capabilities [1–3]. These properties allow a typically small fraction of CSCs to give rise to various lineages of daughter cells, which in turn propagate secondary tumors in primary or distant organs and result in tumor recurrence or metastasis. The existence and roles of CSCs have been under debate due to the variability in the frequency and functionality of CSCs. CSCs can be identified by chosen surface markers, which vary across different cancer types and even subtypes [4–7]. CSC markers only strongly enrich but do not purify the CSC subpopulation within bulk populations of cancer cells. CSC isolation based on the expression of so-called “stem cell marker” failed to distinguish CSCs that exist in the cell-of-origin state or a subset of cancer cells with heightened ability to proliferate. It is not surprising since CSCs are not a fixed but dynamically changing entity. Recent development of two gold standard assays, transplantation and lineage tracing, provides better CSC assessment.* In vivo* tumor transplantation assay is a surrogate assay for evaluating self-renewal and pluripotency of CSCs [8]. Lineage tracing is a method of generic labeling of specific cell types (e.g., CSCs) that is used to determine the potential cell-of-origin of cancer, allowing measurement for long-term clonal growth of CSCs in native environmental niches. In fact, lineage tracing experiments in both colorectal and breast cancer mouse models have proven that CSCs are the origins of these cancer types [2, 9]. Recent studies using these two approaches have reinforced the critical role of CSCs during tumor formation and progression [2, 9]. Similar to stem cells, cell division of CSCs is stimulated upon environmental stress, growth factors, and cytokines. CSCs divide asymmetrically and produce progenitor cells for multilineage cells, allowing for tumor growth and tumor survival. On the other hand, CSCs also produce other daughter cells that retain the slow-cycling and self-renewal traits of CSCs, preserving the CSC phenotype long-term [10–12]. Under stress, CSCs divide into daughter progenitor cells that act as additional tumor-initiating cells which can maintain the primary tumor cell population heterogeneity or form secondary metastases in a more favorable niche [13, 14].

Traditional chemotherapy and radiation treatments are designed to target rapidly dividing cells but are ineffective to CSCs. The three challenges of CSC targeting are the quiescent state of CSCs, small population size, and variable CSC location. The intrinsically quiescent nature of CSCs allows them to escape treatment and persist in the patient as a dormant reservoir for tumor cells [15]. Over time, the residual CSCs can propagate heterogeneous tumors, causing tumor recurrence in patients [16]. Studies have suggested that therapeutic depletion of tumors may result in an even more aggressive cancer due to an increased number of CSCs circulating in the system [17].

The CSC population typically accounts for less than 5% of all cancer cells [18–20]. CSCs can be found inside tumors, in tissues surrounding tumors (due to their highly invasive nature), or circulating through the vascular system [19, 21, 22]. Various locations and small cell amounts make the detection and targeting of CSCs very difficult. Additionally, the CSC state is highly dependent on the signals from the surrounding microenvironment, which specifically influence whether the CSC is self-renewing, differentiating, or regenerating from differentiated cells [23–25]. In particular, the latter event has been proven to be the cause of treatment failures for various types of human cancer [26]. Better understanding of the bidirectional conversion between CSCs and differentiated cancer cells will lead to the development of effective CSC-targeted approaches.

## 2. Epithelial-to-Mesenchymal Transition (EMT) in Human Cancer

Epithelial-to-mesenchymal transition (EMT) was originally discovered for its role during gastrulation of embryogenesis, but more recently EMT activation has been detected in abnormal somatic cells such as cancer cells [27, 28]. In healthy subjects, differentiated epithelial cells form tight cell-to-cell adhesions with neighboring cells, as well as contacts with the basement membrane to compose the epithelium. This continuous layer of cells creates a border that separates the environment's apical and basal surface to the epithelium [27]. This border is dissolved when cells undergo EMT, a process that involves the transcriptional repression of epithelial markers, such as E-cadherin, and expression of mesenchymal markers such as N-cadherin, vimentin, and fibronectin. The resultant mesenchymal cells lose cell-to-cell adhesion and cell polarity and gain migratory and invasive capabilities [3, 29–31]. Positive correlations between EMT-associated genes and poor disease outcomes have been reported in various human cancer types [32–34].

More significantly, EMT reverts differentiated cells back to the stem-like state. It has been shown that the molecular profile of EMT-induced CSCs is similar to that of stem cells [35–37]. Similar to CSCs, mesenchymal cells exhibit greater resistance to traditional therapeutics and the ability to establish secondary tumors after treatment [38, 39]. Following studies that functionally link the EMT process to CSCs have revolutionized the concepts of CSC biology and have drawn attention to the development of EMT-based strategies for targeting CSCs [1, 3, 40]. EMT is a dynamic and reversible process of tumor progression. Therefore, approaches that block EMT by directly targeting genes involved in phenotypic changes of EMT, such as E-cadherin, N-cadherin, and vimentin, are often inefficient [40]. To develop an effective EMT-targeting therapy, better understanding of the molecular mechanisms accounting for EMT activation is critical.

## 3. EMT Transcription Factors (TFs) as Therapeutic Targets for CSC-Based Therapy

**Figure 1:**
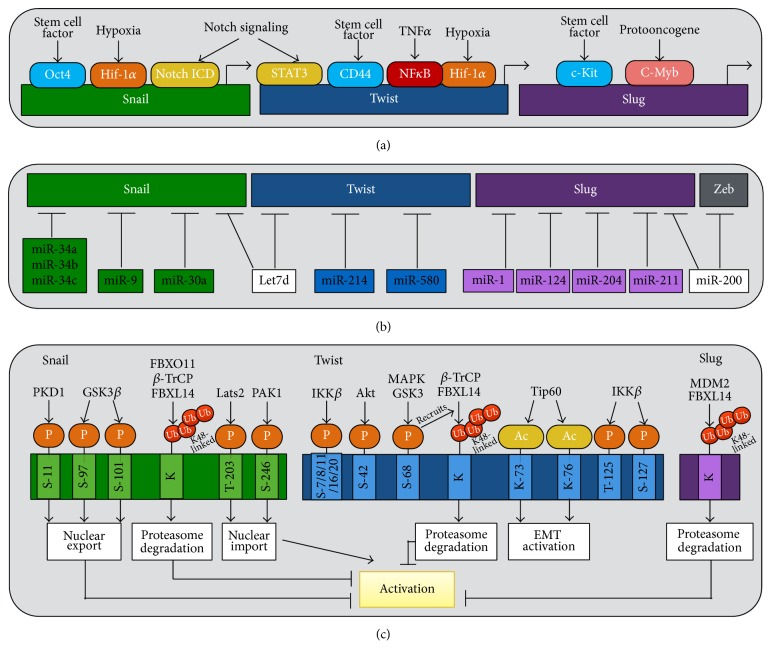
An overview of epithelial-to-mesenchymal transcription factor (EMT-TF) regulation at the (a) transcriptional, (b) translational, or (c) posttranslational level.

EMT activation can be induced by genetic mutations occurring in cancer cells or external environmental stimuli [27]. In both cases, several signaling pathways including transforming growth factor beta (TGF-*β*), Notch, Wnt, and integrin are known to activate EMT through transcriptional repression of E-cadherin [40–42]. E-cadherin functions as a key gatekeeper of the epithelial state. Loss or downregulation of E-cadherin has been considered to be a hallmark of EMT [43]. E-cadherin is mutated or downregulated in various human tumors [29, 31–34]. Apart from the genetic mutation, downregulation of E-cadherin can be mediated by epigenetic silencing as well as EMT-controlling TFs including Snail (Snail1), Slug (Snail2), Twist, zinc finger E-box-binding (Zeb)1/2, and others [44–46]. The Snail and Twist protein families are the most intensively studied EMT-TFs and have been functionally linked to CSC activation [1, 26, 27, 47–49]. Here, we will review the transcriptional, translational, and posttranslational regulation of Snail and Twist (Figure 1) and discuss how these mechanisms contribute to CSC biology.

The Snail/Slug protein belongs to the family of zinc finger TFs, which function to induce EMT through binding to the promoter region of E-cadherin directly or indirectly [50–53]. Snail directly binds to E-box motifs in the promoter region of E-cadherin, represses it, and initiates the EMT process [53]. Snail-mediated E-cadherin repression requires the recruitment of the Sin3A/histone deacetylase 1 (HDAC1)/HDAC2 complex, evident by the blockade of Snail repressor effect by treatment of Trichostatin A, a small molecule compound for selective inhibition of HDAC1 and HDAC2 [54]. Similar to Snail, Slug acts as a direct transcriptional repressor of E-cadherin albeit with weaker binding affinity to the E-cadherin promoter [55]. Ectopic expression of Snail in human mammary epithelial cells endows cells with a mesenchymal phenotype and enriched population of CSCs, promoting CSC-mediated tumor initiation [30]. Conversely, silencing of Snail in breast cancer cells dramatically reduces CSCs, tumor growth* in vivo* and increases sensitivity to chemotherapeutic agents [56]. Likewise, overexpression of Slug has been reported to acquire CSC traits in several cancer types including breast, ovarian, and intestine [57–59]. Slug cooperates with SRY-Box  9 (Sox9) to determine the mammary stem cell state. Coexpression of both TFs promotes the tumorigenic and metastasis-seeding abilities of breast CSCs. Breast cancer patients with primary tumors expressing high expression levels of both Slug and Sox9 are associated with even worse patient outcomes than expression of Slug alone. In line with this observation, induction of EMT program by transient expression of Snail facilitates entrance to stem cell state from luminal progenitors but not from differentiated luminal cells. These findings suggest that in certain scenarios engagement of additional genetic programs, in this case through expression of Sox9, is required for potentiating EMT-TFs' capabilities in entering full CSC state [51].

Twist is a basic helix-loop-helix TF originally shown to be central to embryonic development and later found to be highly expressed in a wide array of metastatic cancers [60]. Further functional analyses establish Twist as a master regulator of cancer metastasis by inducing EMT, increasing tumor cell migration and invasion. Mechanistically, Twist binds to promoter regions and enhance gene transcription of Slug, subsequently leading to gene repression of E-cadherin [61]. Twist can also indirectly repress E-cadherin expression through recruitment of the methyltransferase SET8 that methylates histones for gene silencing [62]. Apart from its EMT-including ability, Twist can work in concert with BMI1, a polycomb-group repressor complex protein, to orchestrate stem cell self-renewal by direct induction of BMI1 gene expression [63]. In view of Twist's versatile roles in regulating cancer stemness and its influence on other EMT-TFs such as Slug, targeting Twist has been considered as a compelling approach for CSC-based therapy.

The Zeb1/2 zinc finger TFs also partakes in the EMT process. Zeb1 gene expression usually follows the activation of Snail. Additionally, Twist has been shown to work in concert with Snail in the induction of Zeb1 [64]. Recent reports connected Zeb1 to cancer stemness. Zeb1 enhanced tumor-initiating properties of pancreatic and colorectal cancer cells by inhibiting the expression of stemness-repressing microRNAs (miRNAs) including miR-200 family and miR-203 [65]. Moreover, Preca et al. have identified a positive feedback loop between Zeb1 and CD44. In breast cancer cells, high levels of the stem cell marker CD44 corresponded to a mesenchymal phenotype by promoting Zeb1 expression. Overexpressed Zeb1 in turn enforced CD44 slicing that favors cancer cells acquiring stem cell features [66]. Mechanisms by which Zeb1 regulates tumor progression, cancer stem cell properties, and chemoresistance are discussed in greater detail elsewhere [67] and thus are not further summarized below.

## 4. Regulation of EMT-TFs

EMT-TFs represent common molecular targets between cancer and CSCs [44, 46]. Aggressive tumor progression and poor therapeutic outcomes have been attributed to characteristic cellular plasticity due to abnormal elevation of EMT-TFs [41]. Expression of EMT-TFs is repressed in somatic tissues but reactivated during cancer development by a variety of cell-autonomous pathways and microenvironmental cues [50]. Molecular mechanisms that control the reactivation of master EMT regulators at different steps of transcription, translation, protein stability, and protein activation are of intense interest.

## 5. Transcriptional Control of EMT-TFs

Gene expression is tightly regulated by TFs to produce cell- and tissue-specific expression patterns. Multiple layers of altered transcriptional regulation can occur during tumor progression including dysregulated TFs related to oncogenesis or through the influence of the tumor microenvironment. Under hypoxic conditions in tumors, stabilized hypoxia-inducible factor 1-alpha (Hif-1*α*) directly induces Snail transcription and subsequent gene repression of E-cadherin [68]. Notch signaling is believed to play a big part in transducing hypoxic stimulus to EMT. Notch signaling deploys two distinct mechanisms that work in concert to regulate the expression of Snail. The intracellular domain of Notch can be recruited to the Snail promoter to induce gene transcription. It can also potentiate Snail stabilization by upregulating gene expression of its stabilizer, hypoxia-induced lysyl oxidase (LOX) [69]. A mouse study with epicardial-specific knockout of the gene encoding Wilms' tumor-1 (Wt1) revealed an essential role of Wt1 in repression of the epithelial phenotype in epicardial cells and during embryonic stem cell differentiation through direct transcriptional regulation of Snail [70]. The involvement of Wt1/Snail axis in tumor-associated EMT has also been confirmed at least in renal cell carcinoma [71]. Stem cell-related TFs have been linked to Snail gene expression. Hu et al. showed that octamer-binding transcription factor 4 (Oct4) appears to facilitate pro-EMT processes via upregulation of Snail in breast cancer cells [72]. By contrary, Li et al. found that Oct4 cooperates with SRY-Box  2 (Sox2) to suppress the pro-EMT signals through downregulating Snail at transcription levels [73]. While these reports implicate Oct4 to Snail gene regulation, future studies will be needed to reconcile the discrete regulation of Oct4 in Snail in different tissues. Similarly, expression of Slug and Slug-mediated treatment resistance attributes to the regulation of stem cell factor c-Kit [74]. Expression of Slug is also controlled by the protooncogene c-Myb in tumor cells of different origins including colon and neural crest [75]. Inversely, several TFs involved in stem cell regulation and development, which include FoxA1, KLF4, Sox3, SIM2, and ELF5, have been shown to directly inhibit Slug gene transcription [76]. Twist acts downstream to a wide array of signaling pathways for mediating tumorigenesis. Elevation of the tumor necrosis factor alpha (TNF*α*) signaling pathway triggers Twist gene expression via recruiting the p65 subunit of nuclear factor kappa-light-chain-enhancer of activated B cells (NF*κ*B) to the Twist promoter region [77]. Activation of Notch induces Twist transcription through signal transducer and activator of transcription 3 (STAT3) [78]. Early animal studies with Twist knockout displays developmental defects reminiscent of genetic loss of Hif-1*α* [79], implicating the interaction between Hif-1*α* and Twist. Indeed, Yang et al. later reported that Hif-1*α*, induced upon hypoxia, can bind to the proximal promoter of Twist for direct activation of Twist transcription, thus promoting EMT and metastatic phenotypes of cancer [80]. Stem cell surface marker and controlling factor, CD44, has been reported to orchestrate Twist gene expression [81]. These findings together depict the engagement of the stem cell machinery in complex regulation of EMT-TFs (Figure 1(a)).

## 6. Translational Control of EMT-TFs

RNA silencing is a conserved gene silencing mechanism in which single-stranded guide RNAs bind to cognate mRNAs and direct their endonucleolytic cleavage or translational repression by RNA-induced silencing complex (RISC) [82, 83]. The ribonuclease type III endonuclease Dicer functions as the key regulator of miRNA biogenesis by processing miRNA precursors into approximately 22-nucleotide noncoding small RNAs. The levels of Dicer tightly control the homeostasis and production of miRNAs. Intriguingly, Grelier et al. reported that Dicer protein expression is reduced in breast cancer with mesenchymal and metastatic phenotypes, accompanied by a global decrease of miRNA expression [84]. This report implicates that miRNA regulates networks in EMT during cancer progression. Several miRNAs have been reported to facilitate EMT via direct repression of Snail. The miR-34 family comprising of miR-34a, miR-34b, and miR-34c is one of the most studied tumor suppressor miRNAs. The miR-34 family is transcriptionally activated by p53 the tumor suppressor that is frequently lost or mutated in a wide array of human tumors [85]. Downregulation of miR-34a/b/c causes upregulation of Snail and subsequently EMT, enhancing migration and invasion. Conversely, Snail binds to E-boxes of the miR-34a/b/c promoters, thereby repressing miR-34a/b/c expression, providing a negative feedback loop in controlling EMT [86]. miR-34a has been shown to suppress CSC self-renewal capacity in breast, prostate, and colon cancer [87]. miR-34a also downregulates stemness factors BMI1, CD44, CD133, OLFM4, and c-Myc [88]. Thus, developing miR-34a as a novel therapeutic agent has been considered as a promising strategy to tackle CSCs. In non-small-cell lung cancer (NSCLC), miR-30a was found to be inversely correlated with invasive potential, upregulation of EMT-associated genes through association with the 3′-UTR of Snail for its gene silencing [89]. In melanoma cells, miR-9 overexpression induced downregulation of Snail with a concomitant increased EMT phenotype via translational repression of NF*κ*B [90]. miR-1 downregulates Slug and such regulation has been functionally linked to EMT, CSC activity, and radio-resistance [57, 91]. Additionally, miR-124, miR-204, and miR-211 have been shown to directly inhibit Slug and revert mesenchymal (or promote epithelial) phenotype in various cell lines [92–95]. Less is known about posttranslational regulation of Twist. miR-214 and miR-580 have been demonstrated to contribute metastatic potentials through translational repression of Twist [96, 97], yet their roles in cancer stemness remain undetermined. Some miRNAs, such as Let-7d and miR-200, can concomitantly repress multiple EMT-TFs. Let-7d represses both Snail and Twist at the posttranscriptional level [98]. miR-200 has been shown to directly target gene expression of Slug and Zeb1/2, another important EMT inducer that has been comprehensively reviewed elsewhere [99], and has been associated with chemoresistance (Figure 1(b)). Development of therapeutics using miRNA mimics of aforementioned miRNAs is highly appealing. For instance, miRNA therapeutics has developed miR-34a mimics which restores expression of miR-34a in tumor tissues and potent antitumor effects of miR-34a mimics have been reported in several mice cancer models [100, 101]. Moreover, the liposomal miR-34 mimic, MRX34, has been developed and tested in Phase I clinical trials in patients with unresectable primary liver cancer.

## 7. Posttranslational Control of EMT-TFs

Posttranslational modification occurs at specific residues of protein substrates. This specificity allows slight modifications to a protein to determine protein fate and localization within the cell. Phosphorylation of Snail has been shown as a critical mechanism regulating its nucleus import and export. Serine- (Ser-) 246 phosphorylation by p21-activated kinase (PAK1) facilitates Snail entry into the nucleus, thereby potentiating its transcription suppressive function on E-cadherin [102]. Large tumor suppressor kinase 2 (LATS2) phosphorylates Snail1 protein at Threonine- (Thr-) 203 which retains Snail in the nucleus and stabilizes Snail protein expression [103]. In contrast, protein kinase D1- (PKD1-) mediated Snail phosphorylation at Ser-11 results in its nuclear export via increased binding affinity to 14-3-3*σ*. Consequently, Ser-11 phosphorylation of Snail blocks EMT and expression of stem cell markers [104]. Additionally, Snail phosphorylation at Ser-97 and Ser-101 by glycogen synthase kinase 3 beta (GSK3*β*) promotes its translocation from the nucleus to the cytoplasm as well as the interaction of Snail with beta-transducin repeats-containing protein (*β*-TrCP) E3 ligases, in turn leading to proteasomal degradation of Snail [105, 106]. At least four kinases including MAPK, Akt, GSK3*β*, and IKK*β* have been reported to orchestrate Twist posttranslationally [107–110]. Twist phosphorylation by MAPK, Akt, GSK3, and IKK*β* reduces Twist protein expression by recruiting F-box and leucine-rich repeat protein 14 (FBXL14-also known as Ppa) and/or *β*-TrCP E3 ligases, which target Twist for Lysine (K)48-linked ubiquitination and subsequent proteasomal degradation [108, 111]. Aside from phosphorylation, Shi and colleagues have shown that Twist is regulated by acetylation at K73 and K76 sites [112]. Acetylation of Twist recruits the BRD4/P-TEFb/RNA-Pol II transcription complex to activate Wnt5a gene expression and subsequent Wnt5a-mediated EMT process, yet it does not affect nuclear transport of Twist. Pharmacological disruption of bromodomain-containing protein 4 (BRD4) binding to Wnt5a promoter by JQ1, a small molecule inhibitor that targets the bromodomain, suppresses EMT, invasiveness, and CSC-like phenotypes in basal-like breast cancer [112].

Ubiquitination (Ub) is a versatile regulatory signal. Protein substrates modified by distinct ubiquitin chains have been linked to specific cellular functions. Ubiquitin chains linked through K48 of the ubiquitin itself are the most abundant and well-studied form of Ub that results in protein degradation and turnover. Snail, Slug, and Twist are highly unstable proteins. Their half-lives are tightly controlled by the proteolytic ubiquitination pathway. Snail phosphorylation by GSK3*β* increases Snail binding to *β*-TrCP for K48-linked Ub as aforementioned [106]. Unlike Snail, Slug lacks the GSK3*β* destruction motif that is essential for interaction and recognition of *β*-TrCP. It is therefore unlikely that Slug stability is directly regulated via the GSK3*β* pathway. Wang et al. have shown that mouse double minute 2 homolog (MDM2) E3 ligase is involved in the protein turnover of Slug [113]. On the other hand, Twist harbors the GSK3*β* destruction motif and is subjected to *β*-TrCP-induced degradation by the proteasome [108]. PKD1-mediated Snail phosphorylation regulates its proteolysis by F-box only protein 11 (FBXO11) and inhibits EMT and metastasis [114]. FBXL14/Ppa is another E3 ligase accounting for Snail degradation [111]. It is unclear whether FBXL14-mediated Snail degradation requires phosphorylation by GSK3*β*. Thus it will be interesting to elucidate whether *β*-TrCP and FBXL14 E3 ligases share the same or contain distinct mechanisms for their interaction with Snail. Of note, the regulation of FBXL14 is not limited to Snail but is common to Slug and Twist [109, 111], implicating a central role of FBXL14 in EMT regulation (Figure 1(c)). Finding ways to stabilize or mimic core E3 ligase FBXL14 will be a potential strategy to disrupt EMT.

## 8. Strategies and Current Advances in Targeting EMT-TFs

Despite a plethora of miRNAs that have been discovered for inhibition of EMT-TFs in preclinical settings, commercial development of miRNA therapeutics is still very limited. The slow progression of miRNA therapeutics stems from the general technical challenges with RNAi-based therapeutics including delivery, stability, and avoidance of activating immune responses [115]. One primary obstacle is the instability of these naked oligonucleotides in biological fluids or tissues. Strategies which include chemical modifications, liposomes, and nanoparticles are employed to improve the half-life and delivery of miRNAs [116]. Since miRNAs regulate many genes, the potential off-target effects of miRNA therapeutics are another major concern. Although systemic delivery of miRNAs could likely target genes in noncancerous tissues, the problem can be solved by tagging miRNA oligonucleotide complexes with antibodies that bind to cancer cells to achieve tissue specificity [117]. With encapsulation in a liposomal nanoparticle formulation, MRX34, a mimic of miR34 and an inhibitor of EMT and CSCs, has successfully entered Phase I clinical trials in 2013 for treating patients with liver cancer (ClinicalTrials.gov identifier: NCT01829971).

Approximately 80% to 90% of protein turnover was mediated by the ubiquitin-proteasome system (UPS). Cancer cells exploit the UPS for their increased growth and decreased apoptotic cell death. The components that make up the UPS represent a diverse group of potential anticancer drugs. Bortezomib is the first-in-class drug designed to target proteasome activity and is FDA approved for the treatment of multiple myeloma. The success of Bortezomib inspired researchers to extensively explore other potential targets of the UPS pathway. E3 ubiquitin ligases are the substrate-recognition protein in the UPS that determine ubiquitination specificity. They are much more specific enzymes compared with ubiquitin activating enzyme E1s, ubiquitin conjugating enzyme E2s and deubiquitinases, and therefore they are potential therapeutic targets [118]. Since FBXL14 is the convergent E3 ligase controlling protein stability of multiple major EMT-TFs including Snail, Slug, and Twist, developing FBXL14 stabilizers might be a plausible therapeutic intervention for CSC targeting via EMT inhibition.

Emerging evidence suggests that different Ub linkages regulate a variety of cellular processes. As discussed above, K48-linked Ub chains are known to regulate protein turnover by signaling a target protein for degradation by the proteasome. In contrast, K63-linked Ub chains account for protein activation. Extensive work has established the vital role of K63-Ub pathway in Akt oncogenic signaling and IKK*β*/NF*κ*B inflammation pathways [119, 120]. A recent report revealed an essential role for nonproteolytic Ub pathway in Twist activation and Twist-mediated EMT and CSC acquisition [121], suggesting that targeting the controlling E3 ligases for K63-linked Ub of EMT-TFs can be a potential approach for development of EMT/CSC-based new cancer therapeutics. It remains elusive whether and how this K63-linked Ub governs protein activity of major EMT-TFs other than Twist. More work will be required to further dissect these possibilities.

## 9. Concluding Remarks 

CSCs and EMT-induced mesenchymal stem cells are associated with poor patient prognosis. These cancerous stem cell phenotypes promote increased invasiveness, metastasis, and notably increased survival even in harsh cellular microenvironments [40]. EMT-TFs have been shown to play a critical role in the acquisition of CSC self-renewal capability and CSC-mediated tumor propagation in xenograft mouse models. However, whether the CSCs derived from EMT induction belong to the group of pluripotent progenitor cells or the cell-of-origin remains largely unknown. Further studies with lineage tracing experiments will be needed for clarification. Nevertheless, recent advances have revealed that transient activation of Twist is sufficient to drive stem cell/CSC phenotypes in skin and breast tissues and moreover, this event is independent of its EMT-inducing activity [122, 123]. Similarly, miR-34a's action in repressing CSC functions is not necessary as a consequence of EMT inhibition [88]. These studies indicate that approaches which inhibit protein expression or activity upstream of EMT-TFs will have a better chance to achieve CSC eradiation. Extensive work as reviewed above shed light on new approaches for the targeting of EMT-TFs. As our understanding of protein regulation of EMT-TFs advances, the ability to generate or repurpose new candidate molecules to target CSCs increases. Specific inactivation of EMT-TFs in combination with chemotherapy will likely enhance patient survival long-term via targeting of both CSCs and differentiated tumor cells. We have reasons for optimism that future studies on structural information of upstream regulators of EMT-TFs and on the crosstalk between upstream regulators and EMT-TFs would yield new CSC therapeutics.
